# Correlation analysis of thyroid function and vitamin D levels in patients with type 2 diabetes

**DOI:** 10.3389/fendo.2025.1650525

**Published:** 2025-08-14

**Authors:** Lizhen Wu, Bo Liang, Ruhai Lin, Zhenfei Fu, Huibin Huang, Jingxiong Zhou

**Affiliations:** Department of Endocrinology and Metabolism, the Second Affiliated Hospital of Fujian Medical University, Quanzhou, China

**Keywords:** type 2 diabetes, vitamin D, thyroid function, correlation analysis, thyroid hormones, HbA1c

## Abstract

**Background:**

This study investigated the association between vitamin D status and thyroid function in 1,805 adults with type 2 diabetes mellitus (T2DM) treated at a tertiary hospital between 2018 and 2024. We analyzed demographic, metabolic, and thyroid function parameters to determine whether vitamin D levels influence thyroid dysfunction in this population.

**Methods:**

Plasma 25-hydroxyvitamin D, thyroid hormones (FT3, FT4, TSH), and autoantibodies (TPOAb, TGAb, TRAb) were measured using electrochemiluminescence. HbA1c was assessed via HPLC. Statistical analyses included Spearman correlation and logistic regression to evaluate relationships between vitamin D levels (categorized as deficient < 20 ng/mL, insufficient 20-29.9 ng/mL, and sufficient ≥ 30 ng/mL) and thyroid disorders.

**Results:**

Vitamin D sufficiency (≥ 30 ng/mL) was positively associated with male sex (OR=2.52), older age (OR=1.05), and higher FT3 (OR=1.28), while inversely linked to elevated triglycerides (OR=0.68) and HbA1c (OR=0.88). Hyperthyroidism showed significant associations with low vitamin D (OR=1.07) and younger age (OR=0.93), whereas hypothyroidism correlated with aging (OR=1.07) and high cholesterol (OR=1.07). No significant relationships were found between vitamin D and thyroid autoimmunity.

**Conclusions:**

Vitamin D deficiency is independently associated with hyperthyroidism and subclinical hyperthyroidism in T2DM patients, suggesting a potential role in thyroid dysregulation. These findings support screening for vitamin D deficiency in diabetic individuals with thyroid dysfunction.

## 1 Introduction

Type 2 diabetes mellitus (T2DM) is a prevalent metabolic disorder characterized by persistent hyperglycemia, primarily resulting from insulin resistance and progressive β-cell dysfunction (1). The increasing global prevalence of T2DM has become a major public health concern, as highlighted by the World Health Organization (WHO) in its reports of a continuous rise in affected individuals worldwide (2). The condition is associated with serious complications, including cardiovascular disease, nephropathy, and neuropathy, all of which substantially reduce patients’ quality of life (3, 4).

Vitamin D, also called 25-hydroxyvitamin D [25(OH)D], a steroid hormone essential for regulating calcium and phosphate homeostasis, plays multifaceted roles in diverse physiological processes, including immune modulation and insulin secretion (5). Vitamin D receptors (VDR) are present in various tissues, such as pancreatic β-cells, immune cells, and the thyroid. Additionally, observational studies have associated low vitamin D levels with numerous chronic diseases, including cardiovascular, autoimmune, and metabolic disorders (6). Emerging evidence indicates that serum 25(OH)D deficiency may impair pancreatic β-cell function, thereby contributing to the development and progression of insulin resistance (7). Specifically, deficiency in 25-hydroxyvitamin D [25(OH)D] has been shown to impair pancreatic β-cell function and promote insulin resistance, hallmark features of type 2 diabetes (T2D) (7–11). Research indicates that maintaining adequate vitamin D levels may improve glycemic control and reduce the risk of multiple T2DM-associated complications (12, 13). In patients with T2DM, vitamin D status has attracted increasing attention due to its potential implications for metabolic health.

Emerging evidence indicates that patients with T2DM demonstrate a higher prevalence of thyroid dysfunction compared to non-diabetic individuals, suggesting a potential pathophysiological link between vitamin D status and thyroid function (14, 15). Additionally, evidence suggests significant differences in both thyroid hormone levels (free triiodothyronine [FT3], free thyroxine [FT4], thyroid-stimulating hormone [TSH]) and thyroid autoantibody levels (thyroid peroxidase antibody [TPOAb], thyroglobulin antibody [TGAb], and TSH receptor antibody [TRAb]) between vitamin D-deficient and vitamin D-sufficient patients with diabetes, potentially indicating an interplay between autoimmune thyroid dysfunction and glucose metabolism regulation (16, 17). Moreover, the high prevalence of vitamin D deficiency in individuals with diabetes raises important questions about its potential association with thyroid dysfunction, particularly regarding thyroid hormone synthesis and autoimmune processes, as evidenced by elevated thyroid antibody levels (18).

Therefore, further investigation is warranted to elucidate the relationships between vitamin D status, thyroid function, and autoimmune thyroiditis in T2DM populations, which could inform the development of targeted therapeutic strategies. Despite these observed associations, studies specifically examining the relationship between vitamin D status and thyroid function in T2DM populations remain limited. This study provided a deeper understanding of these relationships which could inform targeted therapeutic approaches and enhance clinical management in affected individuals.

## 2 Methods

### 2.1 Subjects

This retrospective study analyzed data from 2,495 hospitalized patients with type 2 diabetes mellitus (T2DM) at the Endocrinology Department of the Second Affiliated Hospital of Fujian Medical University between January 2018 and July 2024. Among the participants, 650 had sufficient vitamin D levels (≥30 ng/mL), 1,006 showed insufficient levels (20-29.9 ng/mL), and 839 were deficient (<20 ng/mL). Inclusion criteria required a T2DM diagnosis according to the Chinese Guidelines for the Prevention and Treatment of Type 2 Diabetes (2020 Edition) (19). Exclusion criteria included: (1) acute diabetic complications; (2) pregnancy or lactation; (3) significant cardiovascular/cerebrovascular diseases or severe hepatic/renal dysfunction; (4) history of thyroid surgery; (5) use of medications affecting thyroid function; (6) recent use of vitamin D supplements, glucocorticoids, calcium, or other agents influencing vitamin D levels; and (7) diagnosis of arthritis, recent fractures, metabolic bone disease, severe infections, malignancies, or anemia. The final analysis included 499 patients with sufficient vitamin D (≥30 ng/mL), 736 with insufficiency (20-29.9 ng/mL), and 570 with deficiency (<20 ng/mL).

### 2.2 The criteria used to define thyroid dysfunction

According to established guidelines, the diagnostic thresholds for thyroid dysfunction are defined as follows: Hyperthyroidism is diagnosed when FT3 exceeds 6.8 pmol/L and/or FT4 exceeds 22 pmol/L with concomitant TSH levels below 0.27 mIU/L, while hypothyroidism is characterized by FT3 below 3.1 pmol/L and/or FT4 below 12 pmol/L with TSH levels above 4.2 mIU/L. Subclinical hypothyroidism presents with normal-range FT3 (3.1-6.8 pmol/L) and FT4 (12–22 pmol/L) but elevated TSH (>4.2 mIU/L), whereas subclinical hyperthyroidism shows similar normal FT3 and FT4 levels with suppressed TSH (<0.27 mIU/L). These criteria are further summarized as: (1) hyperthyroidism requires elevated FT3/FT4 with TSH below the lower reference limit; (2) hypothyroidism demonstrates decreased FT3/FT4 with TSH above the upper limit; (3) subclinical variants maintain normal FT3/FT4 but exhibit TSH abnormalities - elevated for hypothyroidism and suppressed for hyperthyroidism.

### 2.3 Vitamin D cutoffs and clinical relevance

#### 2.3.1 20 ng/mL

This threshold for “vitamin D insufficiency” is based on the Endocrine Society Clinical Practice Guideline (2011, J Clin Endocrinol Metab), which states levels <20 ng/mL require supplementation to optimize bone health (Holick et al., 2011, PMID: 21646368). Levels <20 ng/mL are associated with increased PTH secretion and bone loss (Lips et al. Osteoporos Int 2006).

#### 2.3.2 30 ng/mL (75 nmol/L)

This cutoff for “sufficiency” aligns with both the Endocrine Society (≥30 ng/mL for optimal skeletal and extraskeletal effects) and the Institute of Medicine (IOM) report (20–50 ng/mL as safe range; Ross et al., 2011, PMID: 21646369). Observational studies linking levels ≥30 ng/mL to lower risks of falls/fractures (Bischoff-Ferrari et al. BMJ 2009).

### 2.4 Collection of demographic data

The demographic variables collected in this study included gender, age, height, weight, body mass index (BMI, calculated as weight in kilograms divided by height in meters squared), and blood pressure.

### 2.5 Detection of metabolic parameters

Following an 8–10 hour fasting period, comprehensive biochemical analyses were performed using a fully automated biochemical immunoassay analyzer. The assessed parameters included: fasting blood glucose (FBG), total cholesterol (TC), triglycerides (TG), high-density lipoprotein cholesterol (HDL-C), low-density lipoprotein cholesterol (LDL-C), uric acid (UA), alanine transaminase (ALT), aspartate transaminase (AST), blood urea nitrogen (BUN), and creatinine. Plasma 25-hydroxyvitamin D [25(OH)D] levels, fasting C-peptide, and thyroid function parameters were quantified by electrochemiluminescence immunoassay (ECLIA).

### 2.6 Vitamin D reference standards

According to established guidelines (20), serum 25-hydroxyvitamin D [25(OH)D] levels were categorized as follows: <20 ng/mL indicated deficiency; 20-29.9 ng/mL indicated insufficiency; and ≥30 ng/mL indicated sufficiency (21).

### 2.7 Statistical analysis

The data were analyzed using SPSS 20.0 (IBM Corp.). Normally distributed continuous variables were expressed as mean ± standard deviation and compared using independent t-tests. Non-normally distributed variables were presented as median (interquartile range) and analyzed with the Mann-Whitney U test. Spearman’s correlation analysis assessed monotonic relationships between variables. Logistic regression analysis identified factors significantly associated with categorical outcome variables. A p-value <0.05 was considered statistically significant.

## 3 Results

### 3.1 Comparison of baseline characteristics among the three groups

Comparisons among the three vitamin D groups (deficient, insufficient, sufficient) were conducted for demographic and metabolic parameters, including age, sex, BMI, blood pressure (SBP, DBP), lipid profiles (TC, TG, HDL-C, LDL-C), UA, FBG, HbA1c, thyroid function (FT3, FT4, TSH), and fasting C-peptide levels. The analysis revealed statistically significant intergroup differences (p < 0.05) in age, sex distribution, TC, TG, UA, FBG, HDL-C, LDL-C, FT3, FT4, fasting C-peptide, and HbA1c levels (**Table 1**).

**Table 1:** Comparative analysis of basic characteristics among the three groups.

Characteristics	Group1 (vitamin D levels ≥ 30ng/mL)	Group2 (vitamin D levels 20 - 29.9 ng/mL)	Group3 (vitamin D levels < 20 ng/mL)	*P* value
n	499	736	570	
Age(year)	62 (55, 68)	60 (53, 66)	57 (49, 67)	0.000
Sex				0.000
Male	307	333	238	
Female	192	403	332	
BMI (kg/m^2^)	23.80 (21.63, 26.11)	24.03 (21.78, 26.39)	23.78 (21.48, 26.29)	0.587
SBP (mmHg)	129 (120, 140)	127 (119.25, 140)	129 (119, 143.25)	0.075
DBP (mmHg)	80 (75, 87)	80 (74, 87)	80 (74, 88)	0.853
TC (mmol/L)	4.37 (3.64, 5.19)	4.59 (3.79, 5.37)	4.64 (3.81, 5.48)	0.003
TG (mmol/L)	1.29 (0.95, 1.90)	1.43 (1.01, 2.12)	1.60 (1.12, 2.46)	0.000
UA (μmol/L)	315 (264, 372)	304 (245.50, 360.76)	314 (245, 380)	0.048
FBG (mmol/L)	7.46 (6.21, 9.64)	7.73 (6.26, 10.02)	8.28 (6.61, 10.98)	0.000
HDL-c (mmol/L)	1.12(0.94, 1.40)	1.11 (0.92, 1.34)	1.06(0.85, 1.34)	0.002
LDL-c (mmol/L)	2.65 (1.98, 3.41)	2.79 (2.07, 3.56)	2.77 (2.11, 3.58)	0.045
FT3 (pmol/L)	4.48 (4.02, 4.95)	4.36 (3.79, 5.37)	4.04 (3.47, 4.59)	0.000
FT4 (pmol/L)	16.5 (14.9, 18.30)	16.69 (14.79, 18.10)	15.76 (14.00, 18.00)	0.000
TSH (mIU/L)	1.65 (1.12,2.56)	1.87 (1.17, 2.69)	1.89 (1.06, 2.89)	0.119
C-peptide (ng/ml)	3.93 (2.08, 6.21)	3.50 (1.85, 5.83)	3.19 (1.47, 5.27)	0.000
HbA1c (%)	8.00 (6.80, 9.80)	8.70 (7.20, 11.00)	9.10 (7.50, 11.30)	0.000

### 3.2 Correlation analysis between serum vitamin D levels and various factors

Spearman correlation analysis was conducted to examine the relationships between serum 25-hydroxyvitamin D [25(OH)D] levels and various metabolic parameters, including sex, age, body mass index (BMI), blood pressure (systolic [SBP] and diastolic [DBP]), lipid profiles (total cholesterol [TC], triglycerides [TG], high-density lipoprotein cholesterol [HDL-C], low-density lipoprotein cholesterol [LDL-C]), uric acid (UA), glycemic indices (fasting blood glucose [FBG], hemoglobin A1c [HbA1c]), fasting C-peptide, thyroid function (free triiodothyronine [FT3], free thyroxine [FT4], thyroid-stimulating hormone [TSH]), and thyroid autoantibodies (thyroglobulin antibody [TGAb], thyroid peroxidase antibody [TPOAb], TSH receptor antibody [TRAb]). The analysis revealed significant positive correlations between [25 (OH)D] levels and age (r = 0.138, p < 0.001), HDL-C (r = 0.101, p < 0.001), FT3 (r = 0.243, p < 0.001), and fasting C-peptide (r = 0.114, p < 0.001). It should be noted that the apparent correlation with sex (coded as a binary variable) reflects group differences rather than a true linear relationship, suggesting the need for alternative statistical approaches appropriate for categorical variables. Significant inverse correlations were observed between [25(OH)D] levels and TC (r = -0.100, p < 0.001), TG (r = -0.178, p < 0.01), FBG (r = -0.107, p < 0.001), LDL-C (r = -0.068, p = 0.004), and HbA1c (r = -0.172, p < 0.001) (**Table 2**). No statistically significant correlations were found between [25(OH)D] levels and thyroid autoantibodies: TGAb (r = -0.024, p = 0.522), TPOAb (r = -0.051, p = 0.178), or TRAb (r = -0.034, p = 0.374) (**Table 3**).

**Table 2:** Spearman correlation analysis(or Mann-Whitney U test) between serum vitamin D level and clinical factors.

Characteristics	*r/z*	*P* value		*r*	*P* value
Sex	-6.487	<0.001	FBG (mmol/L)	-0.107	<0.001
Age (year)	0.138	<0.001	HDL-c (mmol/L)	0.101	<0.001
BMI (kg/m^2^)	-0.006	0.799	LDL-c (mmol/L)	-0.068	0.004
SBP (mmHg)	-0.031	0.189	FT3 (pmol/L)	0.243	<0.001
DBP (mmHg)	-0.027	0.253	FT4 (pmol/L)	-0.038	0.107
TC (mmol/L)	-0.100	<0.001	TSH (mIU/L)	-0.038	0.107
TG (mmol/L)	-0.178	<0.001	HbA1c (%)	-0.172	<0.001
UA (μmol/L)	0.003	0.899	C-peptide (ng/mL)	0.114	<0.001

The correlation analysis between vitamin D and gender was conducted using the Mann-Whitney U test, while the correlation between other continuous variables and vitaminD was analyzed using Spearman correlation analysis.

**Table 3:** Spearman correlation analysis between serum Vitamin D and thyroid-related antibodies.

Characteristics	r	P value
TPOAb (IU/mL)	-0.024	0.522
TGAb (IU/mL)	-0.051	0.178
TRAb (IU/L)	-0.034	0.374

### 3.3 Association between individual factors and serum vitamin D levels

Logistic regression analysis was performed to assess the association between multiple clinical factors and vitamin D status in patients with type 2 diabetes mellitus (T2DM), with vitamin D levels categorized as the dependent variable. The multivariable-adjusted model included sex as a categorical variable and the following continuous covariates: age, hemoglobin A1c (HbA1c), free triiodothyronine (FT3), total cholesterol (TC), triglycerides (TG), uric acid (UA), high-density lipoprotein cholesterol (HDL-C), low-density lipoprotein cholesterol (LDL-C), fasting blood glucose (FBG), and fasting C-peptide.

For vitamin D-sufficient patients (>30 ng/mL), significant positive associations were observed with male sex (odds ratio [OR] = 2.52, 95% confidence interval [CI]: 1.57-4.03), older age (OR = 1.05, 95% CI: 1.03-1.07), and higher FT3 levels (OR = 1.28, 95% CI: 1.00-1.62), while inverse associations were found with HbA1c (OR = 0.88, 95% CI: 0.79-0.98) and TG levels (OR = 0.68, 95% CI: 0.49-0.95). Among patients with vitamin D insufficiency (20-29.9 ng/mL), significant associations were identified for age (OR = 1.02, 95% CI: 1.00-1.03), FT3 (OR = 1.30, 95% CI: 1.04-1.62), and UA (OR = 0.998, 95% CI: 0.996-1.000) (**Table 4**).

**Table 4:** Binary logistic regression analysis using serum vitamin D levels as the dependent variable.

Dependent variables	Independent variables	B	Wald	OR	95%CI	P value
Vitamin D ≥ 30 ng/mL	Age	0.048	23.125	1.049	1.029-1.070	<0.001
	HbA1c	-0.130	5.779	0.878	0.790-0.976	0.016
	FT3	0.244	3.968	1.277	1.004-1.624	0.046
	TG	-0.381	5.125	0.683	0.491-0.950	0.024
	Sex (male)	0.923	14.798	2.516	1.572-4.026	<0.001
20-29.9 ng/mL	Age	0.018	5.122	1.018	1.002-1.034	0.024
	FT3	0.260	5.298	1.297	1.039-1.619	0.021
	UA	-0.002	4.699	0.998	0.996-1.000	0.03

### 3.4 Association between individual clinical factors and thyroid function parameters

Multivariable logistic regression analysis was conducted to evaluate the association between clinical factors and thyroid function status in patients with type 2 diabetes mellitus. Thyroid function was categorized into five groups as the dependent variable: hyperthyroidism, hypothyroidism, subclinical hypothyroidism, subclinical hyperthyroidism, and euthyroidism (reference group). The model included sex as a categorical variable and the following continuous covariates: age, body mass index (BMI), serum 25-hydroxyvitamin D [25(OH)D] levels, hemoglobin A1c (HbA1c), fasting blood glucose (FBG), fasting C-peptide, total cholesterol (TC), triglycerides (TG), high-density lipoprotein cholesterol (HDL-C), and low-density lipoprotein cholesterol (LDL-C).

The analysis revealed distinct patterns of association across thyroid dysfunction categories. Hyperthyroidism showed significant positive associations with lower vitamin D levels (OR = 1.07, 95% CI: 1.01-1.13) and inverse associations with age (OR = 0.93, 95% CI: 0.89-0.98) and BMI (OR = 0.74, 95% CI: 0.60-0.91). Hypothyroidism was positively associated with age (OR = 1.07, 95% CI: 1.01-1.13) and TC levels (OR = 1.07, 95% CI: 1.01-1.13). For subclinical hypothyroidism, significant inverse associations were observed with HbA1c (OR = 0.88, 95% CI: 0.80-0.97) and male sex (OR = 0.65, 95% CI: 0.44-0.96), while a positive association was found with uric acid levels (OR = 1.004, 95% CI: 1.002-1.005) (**Table 5**).

**Table 5:** Multivariable logistic regression analysis with thyroid function categories as the dependent variable.

Dependent variables	Independent variables	B	Wald	OR	95%CI	P value
Hyperthyroidism	Vitamin D	0.067	5.105	1.069	1.009-1.133	0.024
	Age	-0.068	8.581	0.934	0.893-0.978	0.003
	BMI	-0.304	8.400	0.738	0.601-0.906	0.004
Hypothyroidism	Age	0.068	6.008	1.070	1.014-1.129	0.014
	TC	1.497	4.136	4.469	1.056-18.920	0.042
	Vitamin D	-0.009	0.101	0.991	0.938-1.047	0.750
Subclinical Hypothyroidism	HbA1c	-0.129	6.971	0.879	0.798-0.967	0.008
	UA	0.004	16.628	1.004	1.002-1.005	<0.001
	Sex (male)	-0.431	4.714	0.650	0.441-0.959	0.003
	Vitamin D	-0.015	2.123	0.985	0.966-1.005	0.145
Subclinical Hyperthyroidism	Vitamin D	-0.036	2.859	0.965	0.925-1.006	0.091

## 4 Discussion

The complex interrelationship between type 2 diabetes mellitus (T2DM), thyroid dysfunction, and vitamin D status has emerged as a significant focus of metabolic research, with growing evidence suggesting bidirectional interactions that collectively impact glucose homeostasis. Our study identified a significant association between hyperthyroidism and vitamin D deficiency in the T2DM population, reinforcing the need to examine these connections more deeply. This discussion synthesizes our findings with current literature to elucidate three potential mechanistic pathways: (1) vitamin D-mediated modulation of thyroid autoimmunity, (2) shared metabolic disruptions in calcium-vitamin D-parathyroid hormone axis and glucose regulation, and (3) the synergistic effects of vitamin D deficiency and thyroid dysfunction on insulin resistance.

### 4.1 The role of vitamin D deficiency in insulin resistance

Contemporary research has established that vitamin D’s physiological functions extend far beyond its classical role in calcium homeostasis. The near-ubiquitous expression of the vitamin D receptor (VDR) across human tissues and cell types underscores its pleiotropic effects on various biological systems (22, 23). Multiple longitudinal studies have demonstrated a significant inverse association between serum 25-hydroxyvitamin D [25(OH)D] concentrations and insulin resistance, a core pathophysiological mechanism underlying type 2 diabetes mellitus (T2DM) development. This relationship persists after adjustment for potential confounders including BMI, physical activity, and seasonal variation, suggesting an independent role of vitamin D in glucose metabolism regulation (10, 24, 25). Our findings demonstrate a significant positive association between serum 25-hydroxyvitamin D [25(OH)D] levels and fasting C-peptide concentrations (β = 0.24, p < 0.001), suggesting vitamin D sufficiency may be associated with preserved β-cell function in patients with type 2 diabetes mellitus (T2DM). This observation aligns with emerging evidence that vitamin D modulates both insulin secretion and sensitivity through multiple pathways, including: (1) direct effects on pancreatic β-cell VDR expression, (2) regulation of intracellular calcium flux in insulin-sensitive tissues, and (3) modulation of systemic inflammation. Conversely, we observed significant inverse correlations between serum 25-hydroxyvitamin D [25(OH)D] levels and multiple metabolic parameters: total cholesterol (TC: r = -0.100, p < 0.001), triglycerides (TG: r = -0.178, p < 0.01), low-density lipoprotein cholesterol (LDL-C: r = -0.068, p = 0.004), fasting blood glucose (FBG: r = -0.107, p < 0.001), and hemoglobin A1c (HbA1c: r = -0.172, p < 0.001). These consistent negative associations suggest vitamin D sufficiency may exert protective effects on lipid metabolism and glycemic control in patients with type 2 diabetes. The inverse relationship between serum vitamin D levels and both HbA1c and FBG is consistent with previous studies suggesting that vitamin D deficiency is associated with impaired glucose metabolism and an increased risk of diabetes. We performed logistic regression analyses to evaluate the influence of multiple clinical factors on vitamin D status in patients with type 2 diabetes mellitus (T2DM). HbA1c showed a significant negative association with serum 25-hydroxyvitamin D [25(OH)D] levels (OR = 0.878, 95% CI: 0.790-0.976), suggesting vitamin D may play a role in glucose homeostasis regulation and insulin sensitivity enhancement. Additionally, the observed inverse associations between serum 25-hydroxyvitamin D [25(OH)D] levels and both total cholesterol (TC) and triglycerides (TG) may reflect vitamin D’s regulatory role in lipid metabolism, potentially indicating a protective effect against dyslipidemia. Vitamin D is thought to influence insulin sensitivity through multiple mechanisms, including its involvement in calcium metabolism (26, 27), regulation of the renin-angiotensin system (28), and modulation of inflammation (29). Low serum levels of 25-hydroxyvitamin D have been associated with an increased risk of insulin resistance and *the development of* type 2 diabetes (7). The biochemical pathways through which vitamin D may modulate insulin sensitivity warrant further investigation, particularly regarding the role of chronic inflammation in metabolic dysfunction.

### 4.2 The role of thyroid function in glucose metabolism

This study employed multivariable logistic regression to evaluate clinical factors associated with thyroid function. For subclinical hypothyroidism, the observed inverse association with HbA1c (OR = 0.879, 95% CI: 0.798-0.967) suggests better glycemic control may protect against subclinical thyroid dysfunction development, highlighting the bidirectional thyroid-glucose metabolism relationship. Thyroid hormones are well-established as key regulators of metabolic *processes*, including glucose metabolism. Thyroid dysfunction, particularly hypothyroidism, has been associated with impaired glucose tolerance and increased insulin resistance (30, 31). Conversely, hyperthyroidism may lead to enhanced glucose uptake and improved insulin sensitivity (32, 33). The interrelationship between thyroid function and glucose metabolism underscores the importance of assessing thyroid parameters in patients with type 2 diabetes mellitus (T2DM). Thyroid hormones may also regulate vitamin D metabolism by modulating the expression of enzymes involved in the conversion of vitamin D to its active form, 1,25-dihydroxyvitamin D [1,25(OH)2D] (34).

### 4.3 Interrelationship between Vitamin D and thyroid hormones regulation

In this study, multivariable logistic regression analysis was performed to assess the clinical factors associated with thyroid function. The positive correlation between hyperthyroidism and serum vitamin D levels (OR = 1.069, 95% CI: 1.009–1.133) suggests that adequate vitamin D may contribute to the regulation **of** thyroid hormone. Additionally, the inverse relationship with age (OR = 0.935, 95% CI: 0.893–0.978) indicates that younger individuals are more likely to exhibit hyperthyroidism—a pattern that may be attributable to autoimmune conditions such as Graves’ disease, which predominantly affects younger populations. The observed inverse association between BMI and hyperthyroidism (OR = 0.738, 95% CI: 0.601-0.906) suggests that higher adiposity may confer a protective effect, possibly mediated through metabolic and hormonal mechanisms related to increased fat mass. Emerging evidence suggested vitamin D may play a regulatory role in thyroid function (35). Vitamin D primarily exerts its biological effects through the vitamin D receptor (VDR), which is expressed in various cell types, including thyroid follicular cells. Through VDR activation, vitamin D modulates thyroid cell proliferation, differentiation, and functional activity, thereby regulating thyroid hormone synthesis and secretion (36). In addition, vitamin D enhances the innate immune response through VDR activation. It exhibits immunoregulatory properties by modulating cytokine production, which may contribute to thyroid damage and dysfunction (37). Notably, the study found no significant correlations between serum vitamin D levels and thyroid-specific autoantibodies (TGAb, TPOAb, and TRAb), suggesting that the autoimmune component of thyroid dysfunction may not directly influence vitamin D status. Conversely, thyroid hormone levels appear to affect vitamin D metabolism, revealing a potential bidirectional relationship between thyroid function and vitamin D status. This interplay highlights the importance of simultaneous evaluation of vitamin D status and thyroid function when assessing type 2 diabetes risk. Clinically, it may be prudent to monitor vitamin D levels in patients with thyroid disorders and to evaluate thyroid function in individuals with vitamin D deficiency.

### 4.4 Implications for clinical practice

The findings from numerous studies underscore the importance of addressing vitamin D deficiency and thyroid dysfunction in the prevention and management of type 2 diabetes. Clinicians should consider routine screening of patients for vitamin D deficiency, especially in individuals at a elevated risk for developing type 2 diabetes and thyroid disorders. Furthermore, although vitamin D supplementation may offer clinical benefit in certain cases, the optimal dosage and duration of treatment remain to be clearly established.

### 4.5 Study limitations and future directions

While the existing literature offers valuable insights, several limitations remain. Notably, the predominance of cross-sectional study designs limits the ability to infer causal relationships. Furthermore, many studies include relatively homogeneous populations, which may limit the generalizability of their findings to broader, more diverse populations. Future research should prioritize well-designed longitudinal studies and randomized controlled trials to clarify the causal relationships between vitamin D status, thyroid function, and the development of type 2 diabetes. Elucidating the underlying mechanisms that **link** these factors will be essential for the development of targeted interventions in at-risk populations.

## 5 Conclusion

In conclusion, the interrelationship between vitamin D deficiency, thyroid dysfunction, and type 2 diabetes is complex and multifactorial, warranting continued investigation to better understand its pathophysiological and clinical implications. A deeper understanding of these interactions may contribute to developing more effective prevention strategies and therapeutic approaches for managing type 2 diabetes and related metabolic disorders. Additionally, addressing vitamin D deficiency and closely monitoring thyroid function in at-risk individuals may ultimately improve clinical outcomes in the management of type 2 diabetes.

## Data Availability

The original contributions presented in the study are included in the article/supplementary material. Further inquiries can be directed to the corresponding author.
